# Revealing the Effects of Missense Mutations Causing Snyder-Robinson Syndrome on the Stability and Dimerization of Spermine Synthase

**DOI:** 10.3390/ijms17010077

**Published:** 2016-01-08

**Authors:** Yunhui Peng, Joy Norris, Charles Schwartz, Emil Alexov

**Affiliations:** 1 Computational Biophysics and Bioinformatics, Clemson University, Clemson, SC 29634, USA; yunhuip@g.clemson.edu; 2 Greenwood Genetic Center, Greenwood, SC 29646, USA; jwnorris@ggc.org

**Keywords:** missense mutation, energy calculations, Snyder-Robinson syndrome, spermine synthase, binding affinity charge

## Abstract

Missense mutations in spermine synthase (SpmSyn) protein have been shown to cause the Snyder-Robinson syndrome (SRS). Depending on the location within the structure of SpmSyn and type of amino acid substitution, different mechanisms resulting in SRS were proposed. Here we focus on naturally occurring amino acid substitutions causing SRS, which are situated away from the active center of SpmSyn and thus are not directly involved in the catalysis. Two of the mutations, M35R and P112L, are reported for the first time in this study. It is demonstrated, both experimentally and computationally, that for such mutations the major effect resulting in dysfunctional SpmSyn is the destabilization of the protein. *In vitro* experiments indicated either no presence or very little amount of the mutant SpmSyn in patient cells. *In silico* modeling predicted that all studied mutations in this work destabilize SpmSyn and some of them abolish homo-dimer formation. Since dimerization and structural stability are equally important for the wild type function of SpmSyn, it is proposed that the SRS caused by mutations occurring in the N-domain of SpmSyn is a result of dysfunctional mutant proteins being partially unfolded and degraded by the proteomic machinery of the cell or being unable to form a homo-dimer.

## 1. Introduction

Polyamines are cationic polymers that play multiple important roles in a wide range of cell growth and development processes. [1,2,3]. This study focuses on human spermine synthase (SpmSyn), a protein whose function is to convert spermidine (SPD) into spermine (SPM). The reactant (SPD) and the product (SPM) are both polyamines, which are essential for normal mammalian cell growth and development [4,5]. A previous study has illustrated that mutations in SpmSyn are associated with Snyder–Robinson syndrome (OMIM #309583, SRS) [6,7,8]. Mutations can affect SpmSyn’s dimer and monomer stability and alter the wild-type hydrogen bond network, which is important for the enzymatic functionality [7,9,10]. All of these alterations cause the disruption of SpmSyn function and thus result in an abnormal SPM/ SPD ratio and SRS. SRS is a rare form of X-linked intellectual disability characterized by mild to moderate mental retardation, asthenic body build (marfanoid habitus), diminished muscle bulk, osteoporosis, kyphoscoliosis, dysmorphisms (facial asymmetry, full lower lip, long great toes) and nasal or dysarthric speech [6,8]. Significantly decreased SpmSyn activity results in low levels of intracellular SPM and a decreased SPM/SPD ratio for Snyder–Robinson syndrome patients. SpmSyn is thus an important drug target to restore the protein’s function [10,11].

SpmSyn consists of two structural domains, C-domain and N-domain, connected via a linker-domain. Structural and biochemical analyses have shown that the biological unit of SpmSyn is a homo-dimer instead of a monomer [9,12]. The C-terminal domain is the catalytic domain, which carries out the catalysis of SPD to SPM. In contrast, the N-terminal domain is not involved in catalytic function but plays a crucial role for the dimerization. Most of the binding interface is formed with the N-terminal domain and deletion of the N-domain disables dimerization and results in the lack of activity [4,11]. Missense mutations occurring in SpmSyn can directly affect the wild-type properties of the active site in the C-domain or alter the binding interface in the N-domain to lower dimer affinity [9,11]. Since the SRS is caused by various molecular mechanisms, combined *in silico* and *in vitro* investigations are necessary to reveal molecular effects of missense mutations in SpmSyn in order to identify drug-like small molecules for disease treatment [10,11,13,14].

Here, we investigate the molecular effect of five SRS causing mutations located within the N-domain of SpmSyn: M35R, G56S, F58L, G67E and P112L. Since these mutations are away from the active center of SpmSyn, they are not expected to directly affect the catalytic function of SpmSyn, but rather to alter SpmSyn activity indirectly by perturbing other biophysical properties. Here we focus on two of them, the stability and the dimerization of SpmSyn.

Some of the abovementioned mutations were previously investigated; others are reported in this work for the first time. Thus, M35R was identified at the Greenwood Genetic Center from a patient diagnosed with SRS. The P112L is the SRS-causing mutation included in this work due to personal communication with Raymond family. The G56S (rs121434610), which occurs at a highly conserved residue within the N-domain region of SpmSyn, greatly reduces SpmSyn activity and leads to severe epilepsy and cognitive impairment [15]. The F58L (rs397515549) also greatly reduces SpmSyn activity and leads to mental retardation along with severe osteoporosis [16]. The G67E (rs397515553) causes an ectopic kidney and early-onset epilepsy in addition to features characteristic of Snyder-Robinson syndrome and completely destroys SpmSyn activity in the patient’s lymphoblastoid cells [6].

## 2. Results

### 2.1. Effect of Missense Mutation on Monomer Stability (in Silico Modeling)

Table 1 shows the results of monomer stability changes (changes of the folding free energy) due to missense mutations calculated with webservers and stand-alone computer algorithms. For most of the cases, predictions made with different algorithms are in good agreement. The most controversial prediction is made by FoldX, where F58L is predicted to stabilize the monomer while other tools give opposite results. The five disease-causing mutations are all predicted to decrease monomer stability. Specifically M35R, G67E and G56S are predicted to dramatically decrease monomer stability.

**Table 1:** Predictions of monomer stability change due to missense mutations. The calculated folding free energy changes are in kcal/mol. ∆∆G > 0 indicates stabilization while ∆∆G < 0 indicates destabilization. Average value (AV) of folding free energy changes is given in the last column of the table. Standard deviation (SD) is also calculated to quantify the variation of energy changes.

Mutations	PoPMuSiC	DUET	FOLDX	I-Mutant 2.0	SDM	SD	AV
M35R	−0.93	−0.47	−0.42	−1.81	−2.93	1.06	−1.31
G56S	−1.99	−0.52	−3.50	−2.16	−3.51	1.24	−2.34
F58L	−1.77	−0.95	2.12	−2.72	−0.18	1.84	−0.7
G67E	−1.99	−1.26	−1.36	−0.18	−1.34	0.65	−1.22
P112L	−0.87	−0.06	−0.43	−0.83	−1.04	0.40	−0.65

### 2.2. Effect of Missense Mutation on Monomer Stability (in Vitro Experiments)

**Figure 1:**
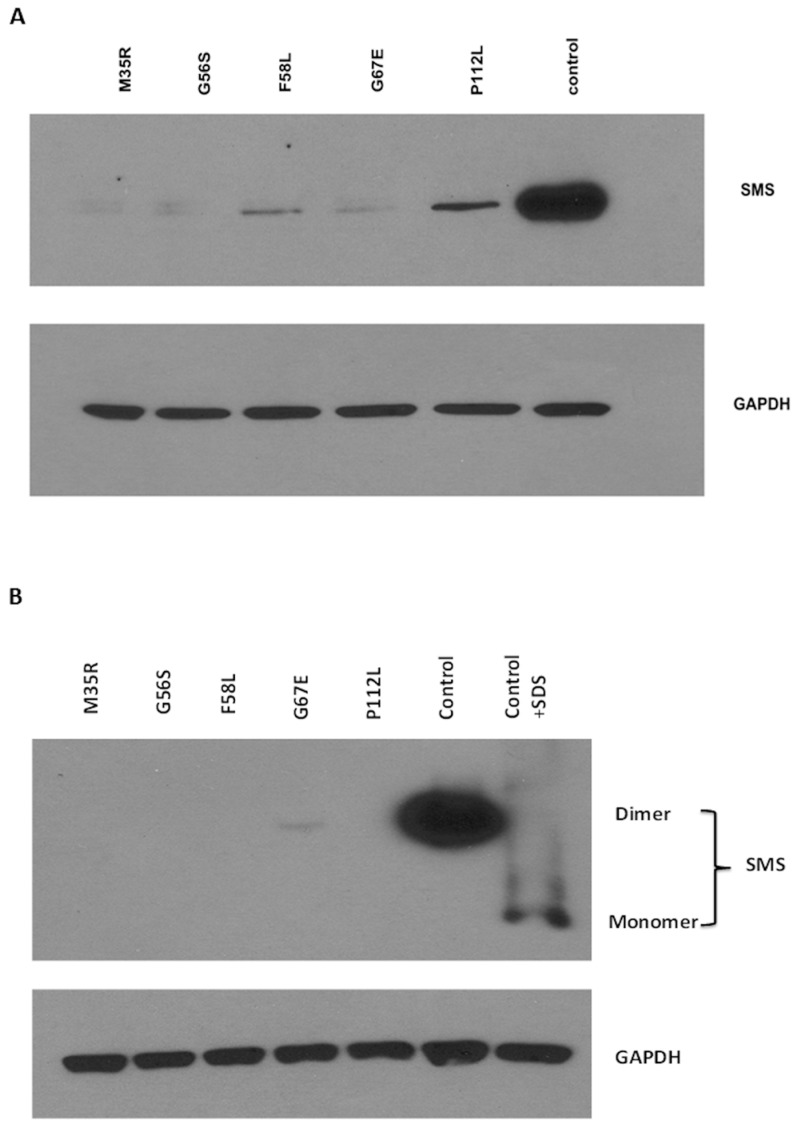
Western blot analysis of SMS levels in patient lymphoblast cell lines. (**A**) Denatured SMS blot. 10 µg of lymphoblast lysate was prepared in Lamelli sample buffer. The buffer was parted on a 4%–20% sodium dodecyl sulfate polyacrylamide gel (SDS–PAGE). Furthermore the buffer was probed for SMS and glyceraldehyde 3-phosphate dehydrogenase (GAPDH). The control was GAPDH. Densitometry of the blots was analyzed using NIH Image J. SMS expression levels of the mutants were normalized to the control; (**B**) Native SMS blot. 10 µg of lymphoblast cell lysate was prepared in native sample buffer, separated on a native PAGE gel and probed for SMS and GAPDH. Densitometry of the blots was analyzed by NIH Image J.

The patient samples showed a reduced level of SpmSyn protein for all the patients either by native or denatured western blot analysis as compared to the control (Figure 1). After native gel electrophoresis, the dimer form of SpmSyn was only detectable in the lane for the G67E alteration. Its level was only detectable upon long exposure. 

On denatured western blots, the P112L alteration was detected at about 20% of the control; F58L was detected at about 7% of the control, and G67E was detected at about 5% of the control. M35R and G56S were barely detectable (Table 2). The implied stability order from this data is: WT > P112L > F58L > G67E > G56S > M35R.

**Table 2:** Densitometric analysis of bands present in denatured gel.

Mutation	SMS/GAPDH Ratio	% Ctrl
M35R	0.04	1.5
G56S	0.07	2.6
F58L	0.18	6.6
G67E	0.13	4.8
P112L	0.55	20.3
Ctrl	2.71	100

### 2.3. Effect of Missense Mutation on Dimer Affinity

Table 3 shows the results for dimer affinity change (binding free energy change) due to missense mutations calculated with webservers and stand-alone computer algorithm. The calculations for all investigated mutations are performed using “AB” dimer and “CD” dimer. For most cases, the predictions made by different algorithms are in agreement. Among disease causing mutations, G56S, F58L, G67E and P112L, are predicted to substantially decrease dimer affinity while M35R is calculated to have negligible effect.

**Table 3:** Predictions of dimer affinity change due to missense mutations. The calculated binding free energy changes are in kcal/mol. ∆∆G > 0 indicates stabilization while ∆∆G < 0 indicates destabilization. The calculation is performed for “AB” dimer and “CD” dimer for comparison. The mean value is the average of all calculated results for each mutation. SD is also calculated to quantify variation of values.

Mutations	BeAtMuSiC (AB)	BeAtMuSiC (CD)	Foldx (AB)	Foldx (CD)	SAAMBE (AB)	SAAMBE (CD)	SD (AB)	MEAN (AB)	SD (CD)	MEAN (CD)
M35R	0.05	0.24	0.17	−0.96	−0.27	−0.19	0.22	0.11	0.60	−0.30
G56S	−1.84	−1.34	−8.64	−11.87	1.58	−4.12	5.20	−5.24	5.45	−5.78
F58L	−2.74	−2.28	−0.1	−1.26	2.20	7.46	2.47	−1.42	5.35	1.31
G67E	−0.78	−0.83	0.372	0.16	−1.86	9.91	1.12	−0.76	5.93	3.08
P112L	−0.11	−0.17	−4.59	−3.32	−0.38	3.39	2.51	−2.35	3.35	−0.03

### 2.4. Result of Multiple Sequence Alignment Analysis(MSA)

**Figure 2:**
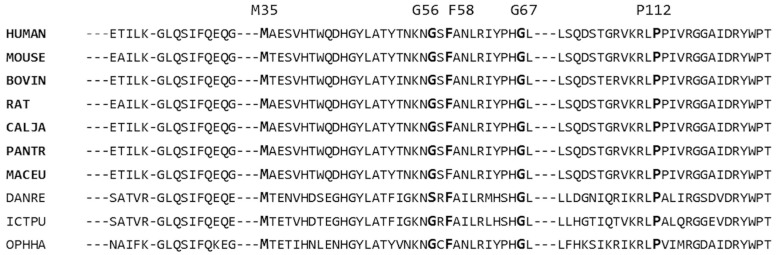
Sequence alignment of SpmSyn among different species. The mutation sites considered in this study are represented in bold letters and the position of the residue in human SpmSyn is shown at the top. The mammals considered in this study are represented in bold letters. Multiple sequence alignment (MSA) is performed with Cobalt Constraint-based Multiple Protein Alignment Tool (COBALT).

We investigated the evolutional conservation of the wild type (WT) residues involved in the mutations based on MSA. The SpmSyn proteins used for MSA are taken from ten different species, which include seven mammals and three non-mammals. Figure 2 shows the result of multiple sequence alignment of SpmSyn among different species. It can be seen that the residues involved in SRS are almost totally conserved across all different species, indicating that these residues are probably important for protein function. The substitution of these highly conserved residues will probably have a large impact on the protein’s functionality. 

## 3. Discussion

**Figure 3:**
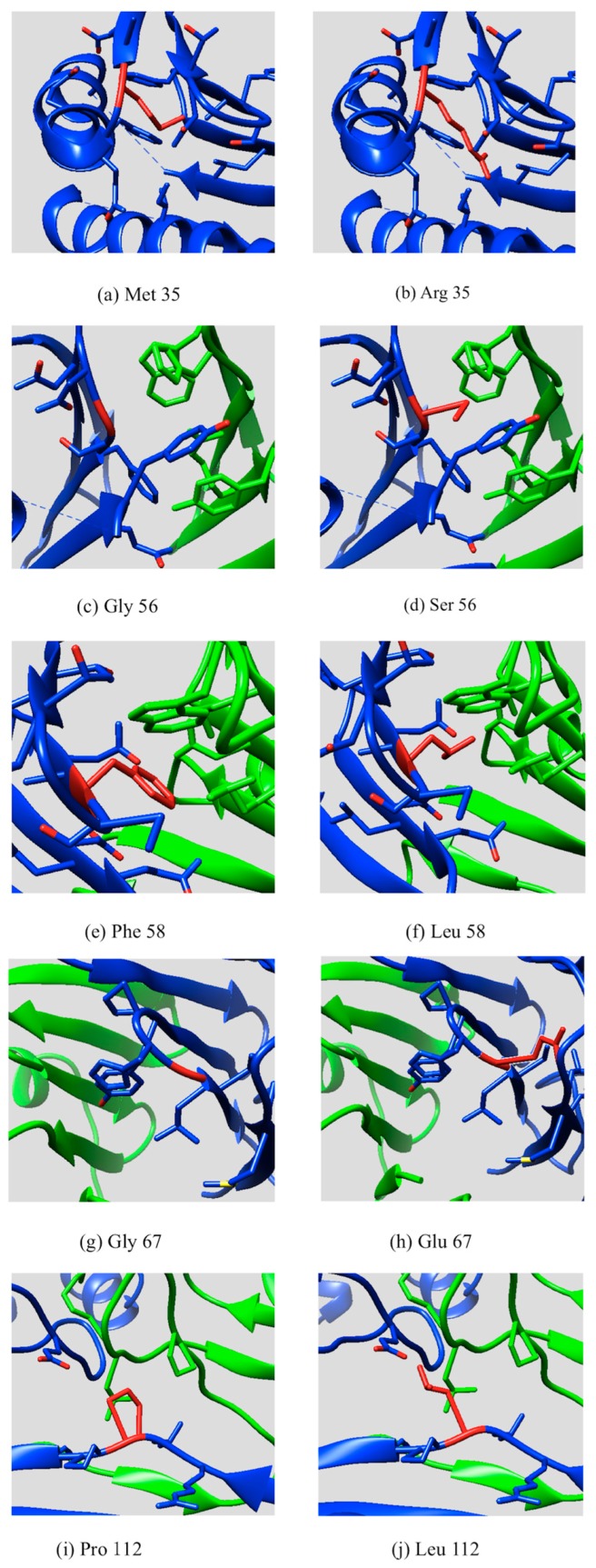
The side chain conformation of five disease-causing mutations mapped onto SpmSyn: (**a**) Part of SpmSyn zoomed at WT position of Met35; (**b**) Part of SpmSyn zoomed at MT position of Arg35; (**c**) Part of SpmSyn zoomed at WT position of Gly56; (**d**) Part of SpmSyn zoomed at MT position of Ser56; (**e**) Part of SpmSyn zoomed at WT position of Phe58; (**f**) Part of SpmSyn zoomed at MT position of Leu58; (**g**) Part of SpmSyn zoomed at WT position of Gly67; (**h**) Part of SpmSyn zoomed at MT position of Glu67; (**i**) Part of SpmSyn zoomed at WT position of Pro112; (**j**) Part of SpmSyn zoomed at MT position of Leu112; The side chain of WT and MT position is shown in red. Two different chains of the dimer are shown in blue and green.

Dimerization is essential for the normal function of SpmSyn and the N-terminal domain plays a crucial role in the dimerization. In this work, we investigated the molecular effect of five mutations which cause SRS located within the N-domain of SpmSyn, focusing mainly on the stability and the dimerization of SpmSyn. To analyze the effects of these mutations on SpmSyn stability and binding affinity, we investigated the structural features of the side chains involved in the mutations and made connections to computational and experimental results. We performed such an analysis for each mutation separately. Figure 3 shows the side chain conformations of the wild type and the mutant residue for the five disease-causing mutations studied in this work.

M35R: The M35R is substitution of a hydrophobic residue Met by a positive charged residue, Arg. The M35 site is totally buried in the protein’s interior. As it is shown in Figure 3a, the structure around M35 site is very well packed and there is no room to accommodate the large Arg side chain. In addition, placing a charged residue, Arg, in the hydrophobic protein interior is energetically very costly and is typically referred as to desolvation penalty. It can be seen (Figure 3b) that the mutant R35 does not establish any hydrogen bonds or other type of favorable interaction. In terms of binding affinity, the M35 site is far away from the interface and a direct effect of the mutation on the binding affinity is not expected. This is consistent with the energy calculations and experimental observation, which showed that M35R has large effect on the monomer stability but negligible effect on the dimer affinity.

G56S: The mutation G56S is located ina sharp loop connecting two β strands of the N-domain (Figure 3c). The G56 site is almost totally exposed to the water in monomer state. However, it is well known that Gly is typically found in tight turns and any replacement may cause steric clashes. This is the structural argument for predicting that a substitution with a relatively long side chain of Ser is not favorable (Figure 3d). The G56 is at the periphery of the dimer binding interface and direct effect on binding is expected for any residue substitution. This is consistent with the energy calculations reported here and in previous work [9,11], showing that the G56S mutation decreases both monomer stability and dimer affinity. The effects were also experimentally confirmed [9]. 

F58L: The mutation F58L is located in a β strand at the dimer binding interface; (Figure 3e,f). The energy calculations predict that the mutation destabilizes the monomer and has a large effect on dimer affinity. The experiments show that the mutation decreases monomer stability as well. As for the dimer affinity, Phe58 is totally buried at the dimer binding interface formed by two β sheets and there is no room for accommodating side chain of different volume—thus the substitution ofLeu is predicted to greatly decrease dimer affinity.

G67E: The mutation G67E is a substitution of a neutral residue Gly by a negative charged residue, Glu, and is located in a sharp loop connecting two β strands of N-domain (Figure 3g). Similar to the effect of the above mentioned mutation G56S, the replacement of Gly by Glu, which is a negative charged residue with long side chains and does not form any favorable interactions (Figure 3h), in a tight turn in structures will cause steric clashes and probably destabilize the protein. This predicted effect is consistent with the energy calculation that G67E significantly destabilizes the monomer stability. The experiments also confirm the computational prediction. In terms of dimer affinity, G67E is at the periphery of the dimer binding interface and the prediction is that G67E mutation will also destabilize the dimer.

P112L: The last disease causing mutation, P112L is also located at the binding interface (Figure 3i,j). The P112 site is totally exposed to the water in the monomeric state. However the substitution is predicted to decrease stability of the monomer. The same is observed experimentally. Furthermore, the P112L is predicted to have a large effect on the dimer affinity as well. It can be seen that P112 site is located at a turn between two β strands and the specific characteristics of Pro residue cannot be mimicked by any other amino acid. This is consistent with the energy analysis indicating that P112L mutation will significantly destabilize the dimer.

From an evolutionary stand-point, the five disease-causing mutations are in sequence positions that are highly conserved across different species in multiple sequence alignment analyses, indicating that they are important for SpmSyn function.

Overall, the study revealed the molecular mechanism of the five SRS-causing mutations: It was shown that all mutations greatly affect SpmSyn stability and dimerization. Thus, the disease-causing effect alters the structural integrity of SpmSyn and thus, the protein is either unfolded, and therefore subjected to degradation, or present in very small quantities with impaired ability to form a dimer. The functional result of either of these events would be a dysfunctional protein resulting in SRS. 

## 4. Materials and Methods

### 4.1. Protein Structure

The wild type structure of human SpmSyn (PDB ID: 3C6K) [12] was downloaded from Protein Data Bank (PDB) [17]. The structure contains four chains and the biological unit is taken as a homo-dimer made of chain “A” and “B” or Chain “C” and “D”. Since there is a small structural difference between the “AB” dimer and the “CD” dimer, both structures are used for energy calculation for comparison. The mutant structure was generated *in silico* by side chain replacement with VMD Mutator Plugin, Version 1.3 [18].

### 4.2. Protein Binding and Folding Free Energy Prediction

Webservers and stand-alone computer programs were applied to assess monomer folding free energy change and dimer affinity change due to mutations. The webservers used for energy calculation included BeAtMusic [19], NeEMO [20], PopMusic [21], I-Mutant 2.0 [22], SDM [23], DUET [24] and CUPSAT [25]. Also a computer algorithm, FoldX 3.0 β3 [26,27] was used to predict the folding free energy changes and dimer affinity change upon single point mutations. Another program developed in our lab, SAAMBE [28], was also used to calculate dimer affinity change.

### 4.3. Multiple Sequence Alignment 

To investigate the evolutional conservation of the above mentioned mutations, multiple sequence alignment (MSA) was performed with Cobalt Constraint-based Multiple Protein Alignment Tool (COBALT) [29] among different species. The sequence of different species was downloaded from UniProtKB/Swiss-Prot Database [30] with FASTA format and include seven mammals: human (Homo sapiens; UniProtKB/Swiss-Prot: P52788), mouse (Mus musculus; UniProtKB/Swiss-Prot: P97355), bovin (Bos taurus; UniProtKB/Swiss-Prot: Q3SZA5), rat (Rattus norvegicus; UniProtKB/Swiss-Prot: Q3MIE9), calja (Callithrix jacchus; UniProtKB/Swiss-Prot: U3FPX7), pantr (Pan troglodytes; UniProtKB/Swiss-Prot: P97355), and maceu (Macropus eugenii; UniProtKB/Swiss-Prot: B3VFB4) and three non-mammals: danre (Danio rerio; UniProtKB/Swiss-Prot: Q9YGC9), ophha (Ophiophagus hannah; UniProtKB/Swiss-Prot: V8NLB9), and ictpu (Ictalurus punctatus; UniProtKB/Swiss-Prot: W5U5T7).

### 4.4. Creation of Lymphoblastoid Cell Lines

Blood was collected from patients into an acid citrate dectrose (ACD) tube and lymphoblastoid cell lines were generatedas previously described: (http://unclineberger.org/research/core-facilities/tissueculture/b-lymphocytesprotocol).

### 4.5. Cell Culture

Patient and control lymphoblast cells were grown in RPMI-1640 media (Thermo Fisher Scientific, Waltham, MA, USA, # MT10040CV) in a 10% humidified CO_2_ incubator. The RPMI-1640 media was supplemented with 15% Fetal Bovine Sera (Atlanta Biologicals, Flowery Branch, GA, USA, # S125450H), 1% antibiotic/antimycotic (Gibco, Grand Island, NY, USA, # 15240-062), and 2 mM l-glutamine (Sigma, St. Louis, MO, USA, # G7513).

### 4.6. Lysate Preparation

For native gels, patient and control lymphocblasts were centrifuged at 450× *g* for 3 min, resuspended in PBS, and centrifuged again for 3 min. The PBS wash was repeated. The cell pellet was resuspended in ice cold 67 mM Tris pH 6.8 with 1× protease inhibitor cocktail (Sigma # P2714) and frozen at −80 °C.

For denaturing gels, patient and control lymphoblasts were centrifuged at 450× *g* for 3 min, resuspended in PBS, and centrifuged at 450× *g* for 3 min. The PBS wash was repeated. The cell pellet was resuspended in ice cold NP40-lysis buffer with 1× protease inhibitor cocktail and frozen at −80 °C. Native and denatured samples were thawed on ice and centrifuged at 10,000× *g* for 10 min at 4 °C. The lysate was quantified by Bradford assay (Waltham, MA, USA, # 23200).

### 4.7. Western Blot Analysis

For a native blot, 10 µg of lysate solubilized in native gel loading buffer (67 mM Tris pH 6.8, 50% glycerol, and bomophenol blue) was separated on a 10% PAGE gel (Bio-Rad, Hercules, CA, USA) without SDS. For a monomer control, the control sample was solubilized in 1× Lamelli buffer but not incubated at 95 °C. For denatured gels, 10 µg of lysate solubilized in 1× Lamelli buffer was separated on a 10% SDS PAGE gel (Bio-Rad). The separated protein was transferred using the Trans-Blot Turbo transfer system (Bio-Rad) to a supported nitrocellulose membrane (Bio-Rad # 162-0090). After transfer, the membranes were rinsed twice with TBST (Tris buffered saline with 0.1% Triton X-100) for 10 min before proceeding to western blotting.

For SMS blotting, the membrane was blocked with block (5% nonfat dry milk), probed with anti- SpmSyn antibody (Abnova, Atlanta, GA, USA # H00006611-M01, 1:2000 in block) overnight at 4 °C, and rinsed three times in TBST for 20 min each. The membrane was then incubated with secondary antibody anti-Mouse IgG HRP (Pierce # 31432 in block), rinsed three times in TBST for 20 min each, and detected on X-ray film after development using the Super Signal West Dura ECL Kit (Thermo Fisher Scientific #PIA34075).

For GAPDH blotting, a duplicate gel was prepared with denatured lysate and blocked with 2% BSA/TBST. The blot was probed with anti-GAPDH antibody (Santa Cruz Biotechnology, Dallas, TX, USA # 32232), rinsed three time in TBST for 20 min each. The membrane was incubated with anti-Mouse IgG HRP (Pierce # 21059), rinsed in TBST three times for 20 min each, and detected on X-ray film after development with the Super Signal West Dura ECL Kit.

## Abbreviations

SpmSyn: Spermine synthase; SRS: Snyder-Robinson syndrome; SPD: Spermidine; SPM: Spermine; GAPDH: Glyceraldehyde 3-phosphate dehydrogenase; MSA: Multiple sequence alignment analysis.
